# Ultra High Throughput Sequencing in Human DNA Variation Detection: A Comparative Study on the *NDUFA3*-*PRPF31* Region

**DOI:** 10.1371/journal.pone.0013071

**Published:** 2010-09-29

**Authors:** Paola Benaglio, Carlo Rivolta

**Affiliations:** Department of Medical Genetics, University of Lausanne, Lausanne, Switzerland; The University of Hong Kong, China

## Abstract

**Background:**

Ultra high throughput sequencing (UHTS) technologies find an important application in targeted resequencing of candidate genes or of genomic intervals from genetic association studies. Despite the extraordinary power of these new methods, they are still rarely used in routine analysis of human genomic variants, in part because of the absence of specific standard procedures. The aim of this work is to provide human molecular geneticists with a tool to evaluate the best UHTS methodology for efficiently detecting DNA changes, from common SNPs to rare mutations.

**Methodology/Principal Findings:**

We tested the three most widespread UHTS platforms (Roche/454 GS FLX Titanium, Illumina/Solexa Genome Analyzer II and Applied Biosystems/SOLiD System 3) on a well-studied region of the human genome containing many polymorphisms and a very rare heterozygous mutation located within an intronic repetitive DNA element. We identify the qualities and the limitations of each platform and describe some peculiarities of UHTS in resequencing projects.

**Conclusions/Significance:**

When appropriate filtering and mapping procedures are applied UHTS technology can be safely and efficiently used as a tool for targeted human DNA variations detection. Unless particular and platform-dependent characteristics are needed for specific projects, the most relevant parameter to consider in mainstream human genome resequencing procedures is the cost per sequenced base-pair associated to each machine.

## Introduction

The recent commercialization of ultra high throughput sequencing (UHTS) technologies, initially applied to the *de novo* characterization of small genomes, is rapidly challenging the classical methods of human genetic research as well. The possibility of obtaining nucleotide sequences in the range of hundreds of millions base pairs from various types of DNA templates allows for example to extend mutational screenings to very large portions of the genome, an experimental strategy that would be too expensive and time consuming to perform with methods based on the Sanger procedure [1]. Thanks to UHTS, intronic and non-coding regions as well can theoretically be included in routine resequencing processes (i.e. the analysis of a DNA region for which a reference sequence is already known) of a particular candidate gene or linkage interval, with minimal additional costs and by a more complete approach with respect to classical exon-PCR and sequencing.

However, these “next-generation” technologies still have some limitations that must be taken into account. A well-recognized problem associated with the mapping of UHTS sequences is represented by the presence of repetitive elements or low-complexity stretches to which short UHTS reads cannot uniquely align [2], [3]. To simplify assembly procedures of short sequencing reads, these DNA segments are therefore generally excluded, with the consequence of missing important disease-associated variants present in intronic or extra-genic areas.

Recently, we discovered a mutation (c.1347+654C>G) in one of these particular regions of the human genome associated with dominant retinitis pigmentosa, an hereditary blinding disease [4]. This single-base substitution is comprised in a repetitive element (variable number of tandem repeats, or VNTR) located within an intron of the *PRPF31* gene. As a proof of concept for UHTS to be used in routine human genetic screenings, we sequenced 31 kb of the human chromosome 19 encompassing the *PRPF31* region in a patient with this rare mutation as well as several common SNPs. For comparative purposes, we used the three currently most widespread UHTS platforms: Roche/454 GS FLX Titanium (Roche 454), Illumina/Solexa Genome Analyzer II (Illumina GA) and Applied Biosystems/SOLiD 3 (ABI SOLiD) instruments.

The Roche 454 technology is based on the clonal amplification of DNA fragments attached on individual beads in an emulsified PCR reaction. The beads are distributed on a 1.6 million wells substrate (PicoTiterPlate™) where pyrosequencing reactions occur [5]. The most noticeable advantage of the Roche 454 platform is the large size of the reads produced (up to 500 nt), while Illumina GA and ABI SOLiD produce shorter reads (34 and 50 nt, at the time this research was performed). In the Illumina GA system the amplification step is achieved on the glass surface that covers the flow cell (bridge amplification) and the sequencing reactions are performed by using the “reversible terminator” chemistry [6]. ABI SOLiD is similar to Roche 454 in the amplification step (emulsified beads) but is unique for its ligase-dependent sequencing chemistry, based on multiple cycles of hybridization and ligation. The main advantage of ABI SOLiD is constituted by the possibility of reading each base twice by independent events, which provides internal error correction and enables higher accuracy, especially in SNP calling [7].

## Materials and Methods

### Ethics statement

This study was carried out in accordance with the tenets of the Declaration of Helsinki and was approved by the Institutional Review Boards of our University and of Harvard Medical School, where the blood was collected and the cell line derived. Written informed consent was obtained from the patient who participated in this study and donated her blood for research.

### Sample preparation

**Figure 1:**
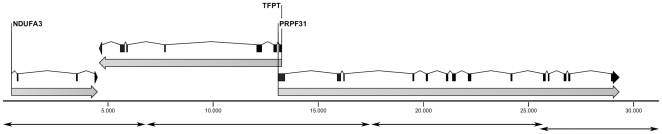
Schematic representation of the 31-kb genomic interval analyzed, containing the genes *NDUFA3*, *TFTP*, and *PRPF31*. Double-headed arrows indicate the position of the 4 amplicons used as template for UHTS.

We extracted DNA from a lymphoblastoid cell line derived from an affected individual carrying the *PRPF31* c.1347+654C>G mutation (cell line #13189) and amplified the 31-kb *NDUFA3*-*PRPF31* genomic region by 4 individually-amplified long-range PCR, designed as previously described [4] (Fig. 1). We specifically selected this region to have a well characterized reference sequence to compare our experimental results to. The following minor modifications were introduced to the original amplification protocol. Each PCR was performed in a final reaction volume of 10 µl, containing 1X GC Buffer I (TaKaRa, Otsu, Shiga, Japan), 0.4 mM dNTPs, 0.2 µM primers (each), 0.5 U of TaKaRa LA Taq (TaKaRa) and 100 ng of DNA. Such an amount of genomic template DNA allows virtually eliminating the possibility that errors introduced by the Taq polymerase are detected in subsequent sequencing procedures. Reactions were incubated at 94°C for 1 min, followed by 35 cycles of 98°C for 5 sec and 68°C for 17 min, and a final elongation step of 72°C for 10 min. After agarose gel analysis and quantification, the four PCR fragments were pooled together and processed for downstream applications.

### Library preparation and sequencing

Preparation of DNA libraries was performed following the guidelines provided by the manufacturers of each platform and sequenced by using: 1/8 of a plate for the Roche 454 Genome Sequencer FLX, Titanium series, 1 lane of an Illumina Genome Analyzer version II, and 1 “quad” of an ABI SOLiD 3 instrument. The exclusion of reads with very low quality was performed automatically by the Roche 454 and Illumina GA sequencing instruments, while for ABI SOLiD this had to be carried out a posteriori with the ABI's csfasta_quality_filter.pl application, available from the SOLiD Software Development Community.

### Alignment and analysis of reads

All analyses and statistics on quality-filtered reads were performed using the relevant tools of the software package CLC Genomics Workbench, version 3.7 (CLC bio, Denmark) as described below.

#### Trimming

In this process the parts of the reads with low quality scores were trimmed. The algorithm calculated base error probabilities based on their quality values, normalized to a PHRED scale. We set a cutoff value of 0.01, calculated as described in the software package manual, and discarded trimmed reads below 20 nt of length, independently from their residual score.

#### Assembly

The original reads as well as the trimmed sets were aligned to 31 kb of the corresponding reference sequence (NC_000019.8: 59,297,572-59,328,826). To ensure uniformity, we applied comparable settings to all platforms, considering the different read length of each platform, inclusive of the color-space option for the ABI SOLiD platform. Specifically, we used the local gapped alignment algorithm for all alignments, keeping the default parameters for mismatch and deletion costs. Reads that aligned to more than one position of the reference sequence were discarded.

For the intronic repetitive DNA fragment we also re-assembled the reads by using a *de novo* assembly procedure. The original reads were first aligned onto the Sanger-obtained sequence of the region by using the same parameters described above and by allowing random matches of reads with multiple mapping positions. Subsequently, we extracted the sequences that aligned to the region and used them for *de novo* assembly with the same parameters used for the reference assembly (no random matches).

#### Detection of variants

Variant detection was performed with the SNP and INDEL detection tools. The settings for calling a variant were described previously by Harismendy *et al.* [8]: if heterozygous, 20%–80% of the reads covering a particular nucleotide had to contain the alternative base with respect to the reference sequence; if homozygous, more than 80% of the reads had to contain the alternative allele. To test the limits of SNP detection, discovery by setting a minimum variant threshold of 10% was also performed. The minimum coverage allowed to call a SNP was of 15 reads for a given base. We applied the default restrictions on SNP calling: the average quality of the central base was set to 20 (PHRED score, corresponding to a base accuracy of 99%), the average quality of the surrounding 10 bases was 20, and the maximum number of mismatches or indels accepted within an 11-nt window was 3. Low quality reads were removed from the calculation of SNP frequency and coverage and the un-aligned parts of a read counted as mismatches. We considered a variant as real if it was validated by Sanger or, in absence of Sanger validation, if it was found in at least one platform and previously annotated, or independently present in at least 2 platforms and not annotated. For the detection of indels we used the same criteria as for SNP detection.

### Coverage simulation

We simulated different coverage depths by randomly sampling a subset of the reads from the *.fastq files exported after the trimming process. Each subset was sampled 3 times. Alignments to the reference sequence were performed as described above for the non-simulated sets of data. In order to have a balanced representation of the 4 amplicons, we calculated the average coverage at the level of the amplicons (and not of the entire region) and joined the amplicons with the same average coverage, considering them as a single (artificial) sampling event. For SNP detection we maintained the same parameters as for the full dataset, but with a minimum coverage of 5 and at least 2 reads carrying the variant allele, to compensate for the reduced coverage introduced by the simulations.

### Sanger sequencing

Data from the Sanger sequencing of the *PRPF31* gene were available from previous analyses [4]. Additional SNPs located outside of the *PRPF31* gene were sequenced starting from the long-range PCRs used as UHTS templates or from short-range PCRs obtained using standard HotStartTaq DNA polymerase (Qiagen, Venlo, The Netherlands) protocols. PCR products were enzimatically purified using 1 µl ExoSAP-IT (USB, Cleveland, Ohio USA) for 10-µl reactions, according to the manufacturer's instructions. Sequencing reactions were performed by mixing 5 µl of purified PCR product, 0.75 µM of 20mer primers and 1 ul of BigDye Terminator v1.1 cycle sequencing kit (Applied Biosystems, Foster City, CA), and run on a ABI-3130XLS (Applied Biosystems).

## Results

### General considerations on the processing and analysis of the reads

All computer-based analyses were performed with a commercial, user-friendly software. This choice was taken in order to be as close as possible to the setup of the average laboratory performing routine genetic testing without the specific support of computer analysts. The use of a simple pipeline, compatible with outputs generated by different sequencing platforms, also allowed treating the data in a uniform manner, thus eliminating possible biases deriving from machine-specific software or algorithms.

### Sequencing and trimming of the reads

For our analyses we used 1/8 of the total sequencing capabilities of each machine. The Roche 454 platform (1 sector of the 8-sector gasket) generated ∼100,000 quality-passed reads with an average length of 318 nt, Illumina GA (1 lane) ∼4.6 million reads of 34 nt, and ABI SOLiD (1 “quad”) ∼17,3 million reads of 50 nt, corresponding to a throughput of ∼32 Mb, ∼157 Mb and ∼862 Mb, respectively (Table 1). We did not consider the option of using paired reads, since this technique would not provide any justified benefits to the analyses made on our standard resequencing project, given the absence of major genomic rearrangement or the necessity of creating a *de novo* assembly.

**Table 1:** Sequence throughput obtained with the three UHTS platforms analyzed.

Sequencing technology	Reads	Count	Discarded reads	Average length of a read (nt)	Bases	Trimmed bases
Roche 454 (1/8)	Total (raw)	99,317		318	31,615,489	
	After trimming	99,317	0.00%	232	23,010,105	27.2%
	After trimming (>20 nt)	98,975	0.34%	232	23,005,448	27.2%
Illumina GA (1 lane)	Total (raw)	4,611,113		34	156,777,842	
	After trimming	4,610,388	0.02%	30	137,405,021	12.4%
	After trimming (>20 nt)	4,245,639	7.9%	31	132,052,133	15.8%
ABI SOLiD (1 quad)	Total (raw)	17,287,756		50	862,377,074	
	After trimming	17,287,610	0.00%	31	534,615,489	38.0%
	After trimming (>201 nt)	13,597,456	21.3%	36	487,053,627	43.5%

All raw sequences underwent quality filtering procedures consisting in the trimming of low quality nucleotides from the reads. After this procedure, 27.2% of the bases from the original throughput were discarded from the Roche 454 dataset, 12.4% from the Illumina GA dataset, and 38.0% from the ABI SOLiD dataset. However, despite the variable number of nucleotides that were rejected, for all platforms the large majority of the reads (>99.8%) were not eliminated, but simply shortened (Table 1, Fig. S1). This outcome changed when, during the trimming procedure, not only the quality of the reads, but also its length was considered. By imposing a minimal size of 20 nt, following the rationale that high-score reads of a few nucleotides are useless for practical resequencing applications, Roche 454 was left with >99.6%, Illumina GA with the 92.1%, and ABI SOLiD with the 78.7% of the original number of reads, corresponding to a loss of 27.2%, 15.8%, and 43.5%, respectively, in terms of nucleotides.

### Alignment to the targeted interval

Trimmed sequences, as well as un-trimmed ones, were mapped to the reference sequence (ref_seq). Since the number and the length of trimmed reads was lower with respect to raw reads, the total amount of bases from trimmed sequences mapping to the ref_seq was also lower. However, trimmed reads mapped to the ref_seq in higher percents, as a consequence of their increased content in high-quality bases, with the effect of producing in principle more accurate consensus sequences (Table 2). These observations were particularly relevant for Roche 454 and ABI SOLiD alignments, rather than Illumina GA, since the latter was less affected by the trimming process.

**Table 2:** Features of reads mapping to the 31-kb reference sequence.

Sequencing technology		Count of Mapped Reads	Mapped reads	Average length of a mapped read (nt)	Total mapped bases	Mapped bases
Roche 454	Full length reads (99,3 K)	62,830	63%	372.85	23,426,369	74%
	Trimmed reads (98,9 K)	78,766	80%	263.45	20,751,152	90%
Illumina GA	Full length reads (4,6 M)	437,1967	95%	34.00	148,646,878	95%
	Trimmed reads (4,2 M)	418,3505	99%	31.14	130,291,345	99%
ABI SOLiD	Full length reads (17,3 M)	14,842,743	86%	49.92	740,988,589	86%
	Trimmed reads (13,6 M)	12,790,106	94%	36.00	460,470,548	95%

**Figure 2:**
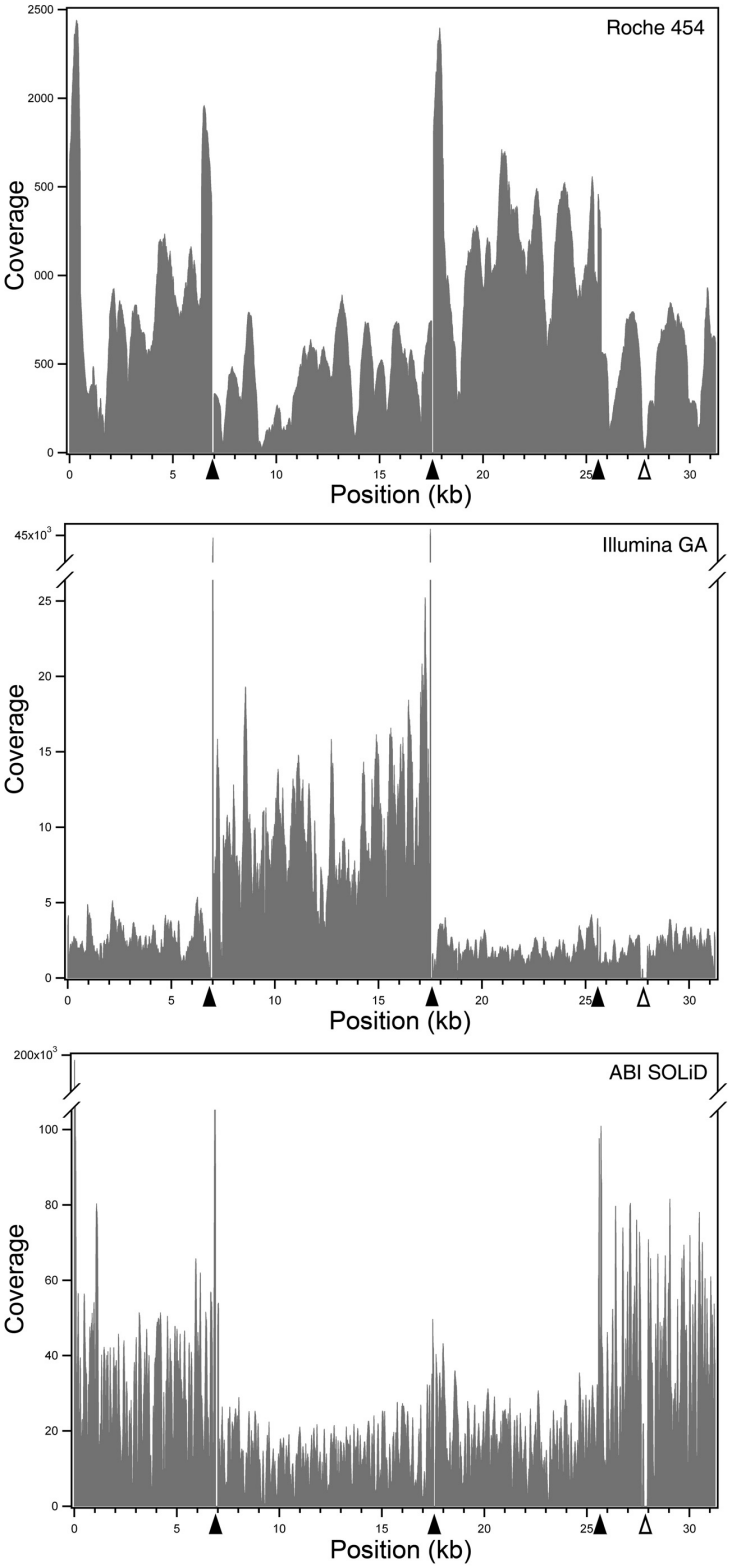
Coverage per bp of the analyzed region, by the assembly of untrimmed reads. Solid arrowheads indicate the boundaries of each long-range PCR amplicon, while open arrowheads show the position of the VNTR in intron 13 of *PRPF31*.

The selected interval was entirely covered using the three datasets, with the exception of 2 very small gaps originating from non-overlapping PCRs (Figs. 1 and 2), a 8-nucleotide gap (position 18,837-44 of the ref_seq) present in the assembled sequence from Illumina GA reads, and 3 small gaps in long homopolymeric stretches in the assembly of Roche 454's trimmed reads (positions 9135-40, 9313-17, 10723-36). The VNTR present in intron 13 of the *PRPF31* gene also presented platform-specific gaps, as detailed below.

Coverage varied depending on the specific LR-PCR product analyzed, because of uneven loading of the individual PCR products (Fig. 2). Similar to the effect of naturally-occurring copy number variants (CNVs) or large-scale deletions, coverage across the analyzed region displayed sudden changes, highlighting at the same time the boundaries between different LR-PCR products. Coverage also varied widely across platforms (Table S1), as a direct effect of the different throughput of the 3 sequencers. High coverage variation also occurred within the same PCR (coefficient of variation for local alignments of untrimmed reads: 0.46 Roche 454, 0.41 Illumina GA, 0.56 ABI SOLiD, Table S1), with a strong bias for the amplicons ends (Fig. 2), a well-known artifact of UHTS [9]. As expected, the average coverage for each amplicon decreased when trimmed sets were used, although it was still much higher than the one required for confident ascertainment of heterozygous genetic variations, estimated by others to be approximately in the range of 10- to 40-fold [6], [7], [10], [11]. In downstream analyses, we kept saturating coverage values to ensure a reliable comparison across platforms and to avoid differences due to stochastic variations of single base coverage.

### Read Accuracy

**Figure 3:**
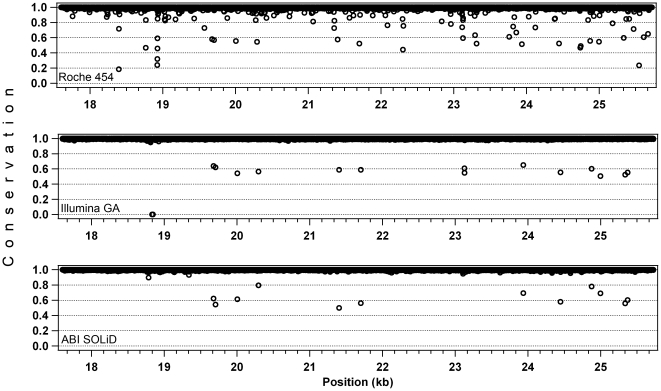
Read accuracy evaluation, on the 3rd long-range PCR fragment (∼8 kb). Values on the Y axis (conservation) indicate the fraction of the most prevalent base at a given position, as detected from the reads covering that position. Conservation scores below 0.5 represent gaps of the assembly with respect to the reference sequence. In the Roche 454 assembly this occurs when the majority of the reads have an indel with respect to the ref_seq, while in the Illumina GA alignment the point at 0% conservation corresponds to a region with no coverage (position: 18,837-44).

To evaluate the accuracy of a base call in each platform after the alignment procedure, we used the “conservation” score, generally used in relationship to alignments of sequences originating from different species. In a resequencing context and as defined by the software package used, this value indicates the percent of the most represented base across the reads covering the same nucleotide in a sequence. An alignment at a given position would have a conservation score of 100% if all the reads carry the same base. For sake of simplicity, to compare the three alignments we selected only one PCR fragment (amplicon #3, ∼8 kb), brought to a simulated average coverage of ∼250x by using sampled trimmed reads. This procedure also allowed evaluating reads that were already filtered by quality scores. The average conservation values were similar across the three platforms (99.38% for Roche 454, 99.56% for Illumina GA, and 99.72% for ABI SOLiD,). However, important differences appeared when values at each position were individually ascertained. In short-read assemblies almost all nucleotides had perfect conservation, with some outliers corresponding to heterozygous SNPs (around 50%). In the long-read assembly the number of outliers was higher, especially within the 80–100% range (Fig. 3). In this latter case, the less conserved positions of the manually-inspected bases were associated with homopolymers stretches and corresponded to either an incorrectly called base or, more frequently, to a gap.

### SNP detection

For comparative analysis of SNP detection performances we considered neither the intronic VNTR containing the *PRPF31* pathogenic mutation, nor another VNTR in the *TFTP* gene, also present in this region.

The number of SNPs identified by setting an allelic threshold of 20% was very similar across all platforms (Table 3). Decreasing the detection threshold to 10% allowed identifying a few more real variants (confirmed by Sanger sequencing), but also 11 more false positives in the Roche 454 and 1 in the Illumina GA datasets, all in correspondence of homopolymeric traits (Table S2). No false positives were detected in ABI SOLiD sequences, even when the threshold was lowered to 10%. Performance in SNP detection was not significantly affected by the use of trimmed vs. raw reads, except for Roche 454 alignments, where the trimming process decreased the number of false positives. This was probably due to the reduction of the coverage below the minimal threshold needed to call a SNP, operated by the trimming procedure itself.

**Table 3:** Number of SNPs and false positive variants detected, after alignments of untrimmed reads.

Sequencing technology	Alignments	True variants >20%	False positives>20%	True variants >10%*	False positives>10%*
Roche 454	Full length reads	48	4	49	15
	Trimmed reads	46	1	47	6
Illumina GA	Full length reads	49	0	52	1
	Trimmed reads	49	0	52	1
ABI SOLiD	Full length reads	48	0	51	0
	Trimmed reads	50	0	51	0

*Values inclusive of the elements detected with a >20% threshold.

For some heterozygous SNPs, mostly located within the 2nd long range PCR fragment, the number of reads relative to one allele was substantially higher with respect to reads belonging to the other one. This effect was particularly visible for the short-read platforms, to a point that the experimental results did not allow a clear detection of the variant, or a clear ascertainment between homozygous and heterozygous SNPs (Table S3). Electropherograms from Sanger sequencing of the same PCR products used as sequencing template for UHTS revealed the same allelic imbalance for some of these SNPs (at positions 7661, 8337, 8564, 9081 of the ref_seq, Table S3). However, when using PCR products obtained by short-range PCR amplification as Sanger sequencing template, electropherograms showed clearly heterozygous peaks for these same SNPs. Taken together, these results may represent the effects of imbalanced amplification of the two alleles prior to sequencing [12], rather then a UHTS-specific or mapping effect.

In all three platforms, the algorithm interpreted the duplication of a CAAG next to an A stretch (dbSNP:5828571) as 2 SNP.

### SNP detection at simulated coverage depths

Coverage simulations were performed to ascertain the presence of features emerging from non-saturating conditions and to determine the minimum coverage required by each platform to detect the correct number of SNPs. We randomly sampled reads after the filtering and trimming procedure to obtain seven average depths (350, 250, 100, 50, 20, 15, and 10x). The average coverage of each fragment was proportional to the number of reads of a given length used in the assembly (data not shown), so that it was possible to calculate the number of reads to sample from each dataset in order to obtain the desired coverage depth.

**Figure 4:**
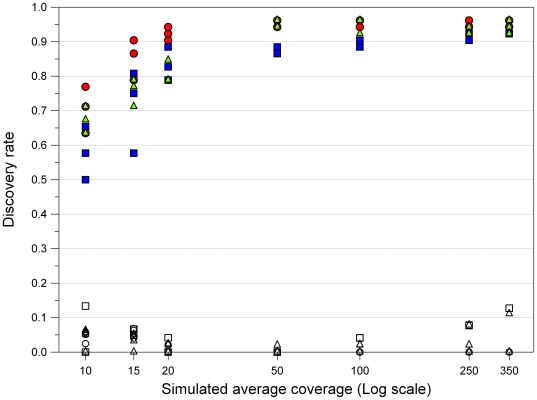
SNP discovery rate at simulated coverage depths. We tested seven average coverage depths, with three random samples for each point. SNPs and false positive hits are indicated by filled and open symbols, respectively. Squares, Roche 454; circles, Illumina GA; triangles, ABI SOLiD.

For each simulated sequencing experiment, we counted the number of SNPs identified. We eliminated all variants detected having less than 5x coverage, allowing at least 2 high-quality reads carrying the variant, since these parameters were already ascertained to produce reliable calls [6]. We chose as “reference set” of detectable SNPs the list of variants reported in Table S3, with the exception of the two entries corresponding to the CAAG duplication (52 SNPs in total). SNP detection following mapping at simulated coverage depths showed some platform-specific differences in the number of variants detected as function of the average number of reads per base (Fig. 4). However, these differences quickly disappeared as soon as the threshold coverage value corresponding to ∼50x was reached. After this limit there was little or no increase in the SNP discovery rate and the different samplings show nearly-similar results. Specifically, at 50x we detected 88% SNPs with Roche 454, and 95% SNPs with Illumina GA and ABI SOLiD, but at higher coverage all platforms reached a plateau score of ∼95%.

False positive appeared in all three platforms. Regardless of the simulated average coverage, they were the outcome of random errors in sequencing that could not be corrected by additional reads covering the same position. At lower depths, this limitation was the obvious effect a reduced number of available reads. At higher depths, false positives invariantly showed to have local coverage that was at least 10 times lower than the average (simulated) one, likely because of mapping difficulties, and thus easily recognizable as false calls.

### Insertions and deletions detection

The automated identification of small insertions and deletions (indels) is a difficult issue both for Sanger sequencing and UHTS technologies. One heterozygous cytosine deletion (dbSNP:34064860) downstream of the *PRPF31* gene was found in alignments for the three platforms. For Illumina GA and ABI SOLiD this was the only indel detected, while for the Roche 454 we could identify 88 (Table S4) and 124 (not shown) additional deletions spanning one to four bp when trimmed and untrimmed reads, respectively, were used. All of them were found in correspondence of homopolymers stretches and were considered as false positives. Moreover, Sanger sequencing of the *PRPF1* gene did not reveal any of the deletions detected by Roche 454 in that interval.

No insertion was automatically found in any of the sequences generated by the three platforms, including the CAAG duplication, ascertained with Sanger sequencing and by manually checking the UHTS alignments (dbSNP:5828571).

### Alignment to the repetitive region of *PRPF31* containing the c.1347+654C>G mutation

**Figure 5:**
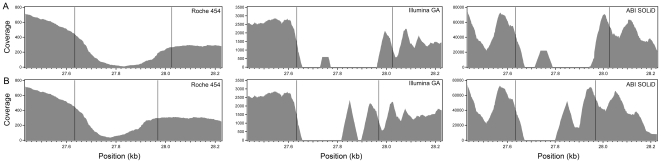
Coverage of the *PRPF31* intronic VNTR. Values shown are relative to the mapping of the original ref_seq entry containing 7 repeats (A) or of the experimentally-determined sequence containing 6 repeats (B). Untrimmed reads are represented here because they produced the best alignment to this repetitive element. Vertical lines show the boundaries of the VNTR.

Repetitive regions represent more than 50% of the human genome [13]. These elements are generally masked in large-scale assembly processes to avoid non-specific alignment of the reads. To overcome this problem, which could have influenced the assessment of variant detection, reads that had multiple matches on the ref_seq were discarded from the analyses. This resulted in lowering the local coverage of low-complexity regions but did not create noise in variant detection. With respect to the VNTR, coverage patterns were not uniform and were platform-specific (Fig. 5A). Unlike reads from Illumina GA and ABI SOLiD, sequences generated by Roche 454 could cover the whole VNTR. Thanks to their longer range, they aligned to the non-repetitive (anchoring) flanking sequences and therefore represented the best option for sequencing this repetitive element. However, the reads deriving from the core repeats had multiple matches and were eliminated, thus resulting in the coverage dip in the corresponding region.

An additional element of complexity typical of repetitive elements such as VNTRs is that they are polymorphic. The individual analyzed here was homozygous for 6 VNTR repeats, while the ref_seq reported a VNTR carrying 7 elements. None of the three platforms analyzed could resolve the correct structure, which was disclosed only by Sanger sequencing. When UHTS reads were aligned onto the correct sequence, short reads assemblies still could not match to the central portion, although the sizes of the gaps were reduced and more sequence could be covered, as consequence of the increased number of uniquely-placed reads. On the contrary, long reads could precisely map the entire region (Fig. 5B). The same occurred also for another VNTR located in the *TFTP* gene: none of the three alignments could clearly detect two repeats present in the patient with respect to the four repeats reported in the ref_seq.

To bypass the limitation arising from forcing an alignment to a reference sequence, we tried also *de novo* assembly of the subset of reads matching the *PRPF31* VNTR (544 Roche 454's reads, 173,824 Illumina GA's reads, 773,064 ABI SOLiD's reads). A *contig* could be obtained only with Roche 454 reads but, as before, the number of the repeats did not correspond to the ones of the patient (one of them was missing).

The mutation associated with adRP in the patient was clearly detected by all three techniques with a frequency very close to 50% and a coverage similar to the rest of the fragment and regardless of the ref_seq used, likely thanks to its proximity with the 5′ anchoring non-repetitive region.

## Discussion

To provide a proof of concept for routine genomic DNA resequencing by UHTS, specifically focused on the detection of disease-causing variants, we processed a 31-kb human genomic region with three next-generation sequencing platforms and analyzed the results with a commercial, user friendly software. In addition to several common SNPs and other typical variants of the human genome, this interval contained a rare mutation located in a particularly challenging region, thus representing an interesting benchmark for a comparative analysis.

The raw sequence throughputs obtained were consistent with the ones expected for the portion of the sequencing area used for each instrument, as specified by each manufacturer. For all platforms, the reads were minimally affected by the filtering (trimming) procedure, as only 0.2% or less of them were discarded. However, this result cannot be taken as a practical qualitative parameter, since reads of excellent quality but of very short length are basically useless in resequencing procedures. When a minimal length of 20 nt was included as a parameter in the filtering process, the three platforms began to reveal some differences. Roche 454 conserved basically all of the original reads, in virtue of its chemistry producing sequences much longer than 20 nt, while the other sequencers retained only 80–90% of them. It has to be noted, however, that this trimming procedure was heavily dependent on the strategies used by the single platforms to eliminate low quality and polyclonal sequences from the raw output and has only a relative value in terms of comparison across the different UHTS systems. For example, ABI SOLiD's low quality reads were not discarded *a priori* by the machine since this platform relies more on quality control steps (color space) during the mapping procedure than during the pre-filtering process.

Following mapping procedures, different platforms produced different coverage depths per base. This was simply the result of the initial different sequencing throughput typical of each platform, and not an issue related to the quality of the sequences or to the mapping procedure. Considering, however, that the same relative sequencing surface was used for all the machines (1/8 of the total sequencing area), mapping of Roche 454 raw reads produced an average coverage of ∼770x/base, of Illumina GA reads ∼4,000x/base, and of ABI SOLiD reads ∼26,000x/base. The throughput of each machine is constantly increasing, following the technical development of the respective chemistries, making it difficult to provide updated comparisons relying on real data analyses. For example, the new released models from Illumina (HiSeq 2000) and ABI SOLiD (version 4) can reach a throughput of 100 Gb per run or more.

Mapping accuracy appeared to correlate with the quality of the individual reads, rather than with parameters related to the mapping procedure itself. Specifically, short-read platforms produced assemblies having higher accuracy than Roche 454, simply because this latter platform is prone to introduce errors (especially indels) when stretches of homopolymeric bases are present [10], [14].

Once the contigs were obtained, we focused on the detection of the human variants contained in the targeted region (SNPs, small insertions and deletions, other polymorphisms), the principal aim being the simulated discovery of pathogenic mutations. SNP detection was overall comparable across the three platforms; however, some differences could be detected. In Roche 454's long-read alignments, false positives and negatives (undetected SNPs) could be again connected to the typical errors of the 454 technology, related to homopolymer effects. We observed that the use of quality-trimmed reads could reduce these false positive calls, but it also reduced the number of true variants automatically detected. Nevertheless, when manually inspected, these variants could safely be identified. Similarly, in alignments from short-read platforms false negatives (one of which was in common between Illumina GA and ABI SOLiD) were due to the low frequency displayed by the “non ref_seq” allele, and they become detectable when the discovery threshold was lowered. In some particular instances, especially for Illumina GA data, the under-detection or the incorrect calling of SNPs as homozygous or heterozygous variants were not a consequence of UHTS errors, but could be explained by allelic unbalanced amplification. This phenomenon occurs when one of the two alleles is enriched during the PCR amplification of the template DNA, or perhaps during the amplification of the libraries, and results in a problem that is relevant also when high coverage depths are used [8]. Notably, in Roche 454 this unbalance was present but less pronounced, indicating probably an inferior sensitivity to this phenomenon. Another interesting observation is that some of the SNPs with low-limit frequency in short-read alignments were located in regions that presented similarities with other segments of the analyzed interval (Fig. S2). One hypothesis could be that some of the reads sequenced from a particular SNP were mistakenly aligned to other similar sequences and vice-versa, lowering the frequency of detection at the real position. Yet, in other regions of similarities SNPs were correctly identified, leading to the notion that errors in allelic calling due to sequence repeats may not represent an absolute rule, especially if the noise is reduced by eliminating reads displaying multiple matches. Taken together, these results indicate that, despite the fact that UHTS machines produce quantitative results, other causes may influence the detection of heterozygous variants when standard parameters are chosen in automated detection. However, in practical terms this issue should not represent a major concern, as the number of SNPs that were prone to this miscalling represented in our test only a small fraction of the total number of heterozygous SNPs.

Sensitivity in SNP detection with respect to the coverage increased from Roche 454 to ABI SOLiD and finally to Illumina GA. Since the differences detected were not too pronounced and SNP detection was heavily dependent on the regional sequence context, we can safely conclude that all platforms analyzed can be considered as having similar performances with respect to sensitivity at the same average coverage. Indeed, it is very hard to extrapolate the results from their specific sequence or random coverage contexts, as the mapping procedure (and the corresponding local coverage of a given SNP) was influenced by the complexity of the DNA to be sequenced and the number of reads available. At lower average coverage depths, the rate of discovery decreased sensitively and different random samplings gave different results because the number of poorly-covered regions was higher. As mentioned, in correspondence of false positive calls local coverage was low even when the average coverage depth was high, indicating a direct influence of the mapping procedure on automated identification of variants.

With respect to detection of small insertions and deletions, the most relevant observation relates to the identification of a large number of false positive deletions in homopolymers stretches obtained with Roche 454 alignments, as also noted by others in analyses of longer genomic intervals [8]. Considering the importance of indels in human hereditary diseases, our experiments indicate that Roche 454 sequences would require the use of specific downstream algorithms, able to systematically detect the presence of sequence-dependent false positives.

The c.1347+654C>G mutation in the 56-bp intronic VNTR of *PRPF31* was taken as a benchmark to assess whether “difficult” DNA variants could be detected by UHTS. Large-scale sequencing projects almost invariantly clash with the problem of mapping and carefully analyzing repetitive DNA elements [2], [3]. Roche 454's long sequences (and presumably any newer UHTS chemistry or technology producing extended reads) represent without doubts the best tool for covering repetitive regions, at least for elements that do not exceed in size the average length of ∼1.5 to 2 reads. Our results support this assumption, since the Roche 454 reads provided the most complete coverage of both the *PRPF31* and *TFPT* VNTRs analyzed. Nevertheless, it was not possible to precisely resolve the number of repeats composing these elements, neither by aligning them to a reference sequence, nor by *de novo* assembly.

Conversely, despite the presence of repeats, all three platforms tested could successfully detect the mutation associated with the disease in the patient's genome. This favorable outcome is probably due to the presence of the pathogenic variant within the first of the 6 elements composing the VNTR, thus allowing the “anchoring” of some reads to the non-repetitive DNA region in 5′ of this repeat. Although previous attempts to identify this mutation with an earlier version of the Illumina GA (the “GA I” platform) failed in such a task [4], this can be explained by the algorithms used for aligning Illumina reads, rather than by the improvements made by the Solexa technology. Specifically, all software used previously allowed random alignment of reads having multiple matches, thus creating noise in the detection of the variant in nearly-identical repeats.

Other rearrangements of the human genome characterized by a variable number of large and unique DNA copies (CNVs, large duplication and deletions, genetic amplification in cancer, etc.) are in general easily detected by UHTS. Because of the quantitative nature of the sequencing results, such rearrangements produce very noticeable variations of coverage when aligned to a ref_seq. For example, CNVs, sparse and non-repetitive elements spanning kilobases to megabases of DNA [15], are simply detected as sudden variations of the coverage by all UHTS platforms analyzed here [7], [11], [16], [17].

The increase of read length in UHTS platforms, an issue on which manufacturers are putting constant efforts, will probably help reducing some of the current weaknesses of this technology and accelerate the transition from Sanger sequencing to UHTS. Illumina, for example, has increased the length of the reads from 35 nt to 100 nt in less than a year; ABI SOLiD, from 50 to 75 nt. However, if we exclude repeats-related concerns, our data seem to indicate that this ever changing dimension in UHTS systems should not have a major impact on DNA variants detection in resequencing efforts (since the reference sequence is known already), whereas the quality of the reads produced should. Hence, the data produced here can very likely be extrapolated to future longer reads from the same platforms, provided that the sequencing chemistry and procedures remain the same.

In our analysis we did not consider the costs of sequencing as a comparative parameter, although it obviously represents an important factor to be taken into account while designing a sequencing project. From our results, no striking qualitative difference appeared across the three platforms, when appropriate conditions in terms of reads and coverage depths were fulfilled. As a general rule then, the less expensive platform producing the needed amount of sequences for a given project would probably also be the most suitable one, unless platform-specific characteristics (e.g. long reads, usable throughput, etc.) are critical for the tests to be carried out or other endeavors with respect to genomic DNA resequencing (e.g. transcriptome sequencing) are performed.

In conclusion, in our work we show that identification of DNA variants in complex DNA sequences such as the human genome can be achieved by highly-parallel techniques, with investments in terms of cost and time that represent a fraction of what is usually spent for conventional sequencing. Furthermore, our successful adoption of a user-friendly software and a straightforward analytical pipeline demonstrates that a strong bioinformatic background is not a compulsory requirement for investigators dealing with UHTS technology. In our example, we performed the analysis of a large genomic region from a single individual amplified by LR-PCR. However, the power of UHTS can be applied to sequence shorter DNA regions obtained by sequence capture or conventional PCR in multiple patients, i.e. to a procedure that is more similar to current routine setups in medical genetic laboratories. Although some limitations to this latter UHTS application still exist, the use of sample pooling [18], [19] and individual DNA barcoding [20], [21], [22], [23] is now facilitating the adoption of highly-parallel sequencers by conventional genetic labs. Taken together, our data indicate that the so-called “next-generation” sequencing, regardless of the platform used, can be efficiently and safely used by the current generation of human geneticists as well.

## Supporting Information

Figure S1 Distributions of read lengths from the three platforms tested. The output generated from short-read platforms consists in reads having the same length: Illumina GA generated only reads of 34 nt and ABI SOLiD generated mostly reads of 50 nt, with only a small fraction of them (0.4%) having shorter lengths.
(0.61 MB TIF)

Figure S2 Similarity plot of the region analyzed. The VNTRs within the *TPFT* and *PRPF31* sequences are indicated by arrows. Vertical lines (corresponding horizontal lines are omitted) indicate the position of SNPs rs35705606 and rs2668836 at coordinates 13,761 and 14,098, respectively, that were under-detected by short read platforms.
(7.67 MB TIF)

Table S1 Coverage of individual amplicons.
(0.05 MB DOC)

Table S2 Details on false positive results detected, after assembly of untrimmed reads.
(0.06 MB DOC)

Table S3 SNPs detected after mapping of UHTS untrimmed reads. Black: SNPs detected by using the default threshold for heterozygozity (20%). Red: SNPs detected with a threshold between 10% and 20%. Blue: SNPs with a borderline limit definition of homo-heterozygosity. Green: variants corresponding to the CAAG insertion. Grey shadow: SNPs located in the 2nd long range PCR fragment, showing allelic imbalance. SNPs identified in the tandem repeats are not reported.
(0.22 MB DOC)

Table S4 Deletions detected with Roche 454 (false positives), using trimmed reads. The deletion at position 30,672 was also found using the other 2 platforms, likely being the only real small deletion.
(0.16 MB DOC)
