# Supplementary figures and images for: Ultra High Throughput Sequencing in Human DNA Variation Detection: A Comparative Study on the NDUFA3-PRPF31 Region
Source: PLoS One. 2010 Sep 29;5(9):e13071. doi: 10.1371/journal.pone.0013071 (PMC2947511; doi:10.1371/journal.pone.0013071)

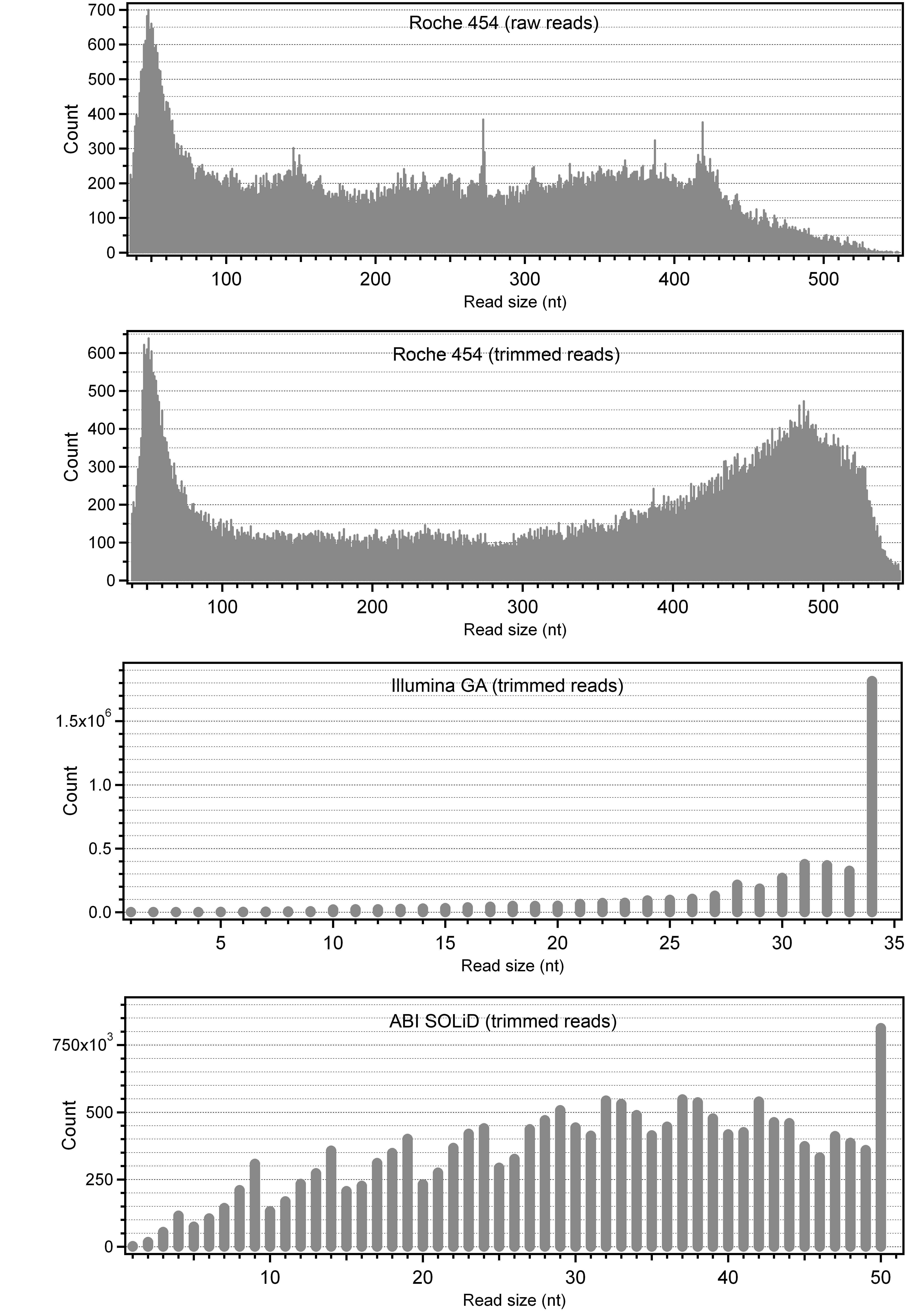

Supplement: Figure S1 — Distributions of read lengths from the three platforms tested. The output generated from short-read platforms consists in reads having the same length: Illumina GA generated only reads of 34 nt and ABI SOLiD generated mostly reads of 50 nt, with only a small fraction of them (0.4%) having shorter lengths. (0.61 MB TIF) [file pone.0013071.s001.tif]

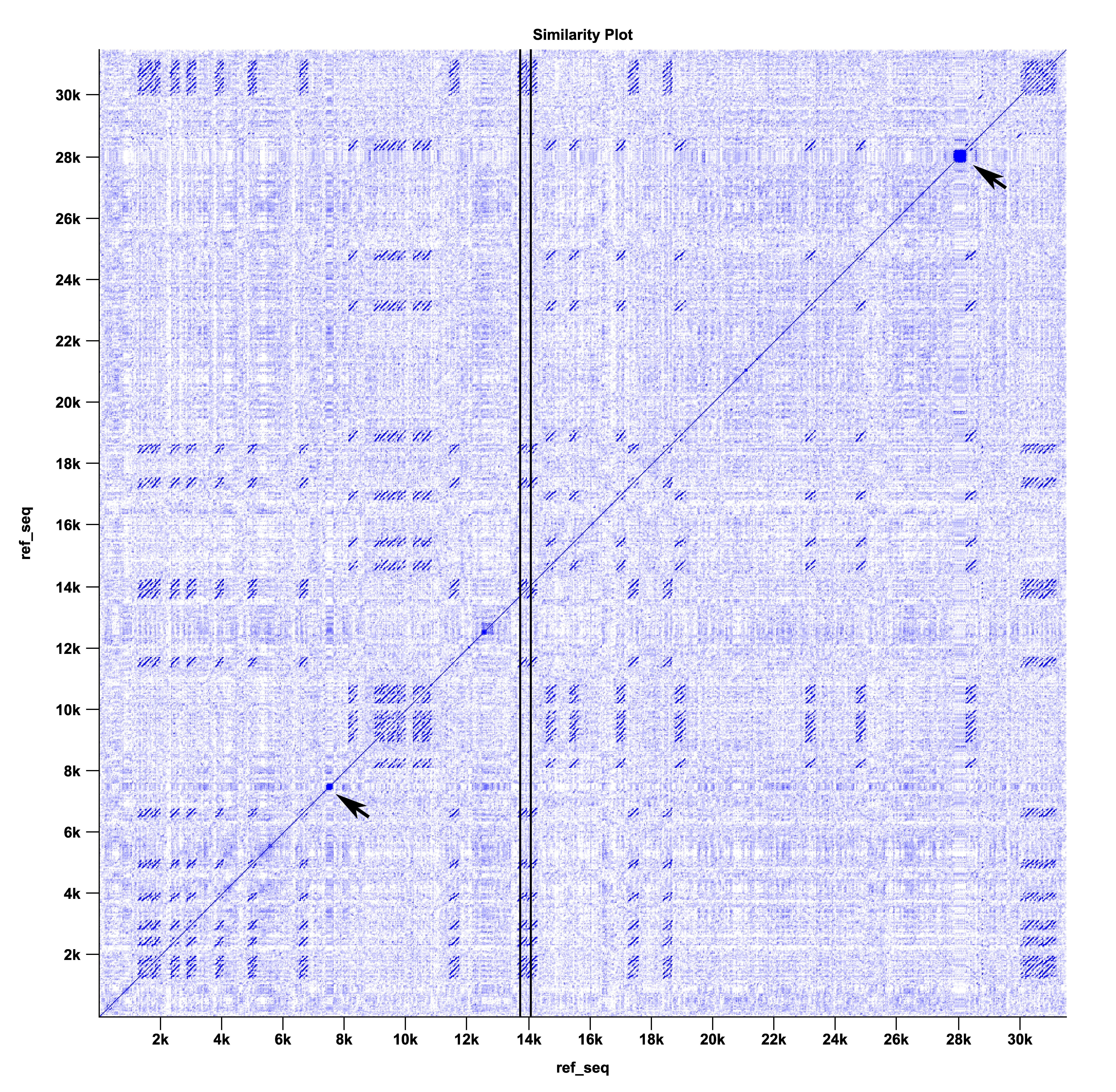

Supplement: Figure S2 — Similarity plot of the region analyzed. The VNTRs within the TPFT and PRPF31 sequences are indicated by arrows. Vertical lines (corresponding horizontal lines are omitted) indicate the position of SNPs rs35705606 and rs2668836 at coordinates 13,761 and 14,098, respectively, that were under-detected by short read platforms. (7.67 MB TIF) [file pone.0013071.s002.tif]
