# Supplementary material for: Ultra High Throughput Sequencing in Human DNA Variation Detection: A Comparative Study on the NDUFA3-PRPF31 Region
Source: PLoS One. 2010 Sep 29;5(9):e13071. doi: 10.1371/journal.pone.0013071 (PMC2947511; doi:10.1371/journal.pone.0013071)
Supplement: Table S1 — Coverage of individual amplicons. (0.05 MB DOC) [file pone.0013071.s003.doc]

|  |  | **Assembly of raw reads** | | | | **Assembly of trimmed reads** | | | |
| --- | --- | --- | --- | --- | --- | --- | --- | --- | --- |
| **Sequencing technology** | **Amplicon** | **Median coverage** | **Mean coverage** | **Coefficient of variation*** | | **Median coverage** | **Mean coverage** | **Coefficient of variation*** | |
|  |  |  |  |  | Mean |  |  |  | Mean |
| Roche 454 (1/8) | 1 | 821 | 916 | 0.56 | 0.46 | 727 | 786 | 0.56 | 0.48 |
|  | 2 | 464 | 438 | 0.48 |  | 420 | 408 | 0.51 |  |
|  | 3 | 1172 | 1188 | 0.31 |  | 1039 | 1050 | 0.34 |  |
|  | 4 | 583 | 541 | 0.49 |  | 497 | 479 | 0.50 |  |
| Illumina GA (1 lane) | 1 | 2391 | 2560 | 0.36 | 0.41 | 2082 | 2250 | 0.37 | 0.40 |
|  | 2 | 9174 | 9823 | 0.46 |  | 7888 | 8635 | 0.48 |  |
|  | 3 | 1777 | 1894 | 0.38 |  | 1539 | 1663 | 0.40 |  |
|  | 4 | 2043 | 1955 | 0.44 |  | 1794 | 1776 | 0.37 |  |
| ABI SOLiD (1 quad) | 1 | 32332 | 33266 | 0.61 | 0.56 | 19672 | 21074 | 0.69 | 0.64 |
|  | 2 | 13569 | 13846 | 0.52 |  | 8202 | 8861 | 0.65 |  |
|  | 3 | 17368 | 18998 | 0.58 |  | 10614 | 11912 | 0.62 |  |
|  | 4 | 37036 | 37580 | 0.53 |  | 21700 | 22971 | 0.59 |  |

*The coefficient of variation for each amplicon is calculated as the ratio of the standard deviation to the mean.
