# Supplementary material for: Ultra High Throughput Sequencing in Human DNA Variation Detection: A Comparative Study on the NDUFA3-PRPF31 Region
Source: PLoS One. 2010 Sep 29;5(9):e13071. doi: 10.1371/journal.pone.0013071 (PMC2947511; doi:10.1371/journal.pone.0013071)
Supplement: Table S2 — Details on false positive results detected, after assembly of untrimmed reads. (0.06 MB DOC) [file pone.0013071.s004.doc]

|  | **Ref_seq pos.** | **Alleles** | **Frequencies** | **Coverage** | **Count of 2^nd^ allele** | **Gene** |
| --- | --- | --- | --- | --- | --- | --- |
|  |  |  |  |  |  |  |
| Roche 454 at 20% threshold | 17133 | C/T | 61.5/38.5 | 78 | 30 | *PRPF31* |
|  | 23116 | A/C | 67.9/28.6 | 28 | 8 | *PRPF31* |
|  | 29924 | T/C | 66.7/33.3 | 15 | 5 |  |
|  | 29925 | T/C | 78.3/21.7 | 23 | 5 |  |
|  |  |  |  |  |  |  |
|  |  |  |  |  |  |  |
| Roche 454 at 10% threshold | 1008 | A/G | 82.1/17.9 | 28 | 5 | *NDUFA3* |
|  | 2307 | T/G | 87.7/10.8 | 65 | 7 | *NDUFA3* |
|  | 3758 | T/G | 81.2/15.6 | 32 | 5 | *NDUFA3* |
|  | 3764 | T/G | 89.5/10.5 | 38 | 4 | *NDUFA3* |
|  | 6610 | T/A | 80.6/16.1 | 31 | 5 | *TFPT* |
|  | 7260 | C/A | 88.5/11.5 | 139 | 16 | *TFPT* |
|  | 18939 | A/G | 80.0/17.8 | 45 | 8 | *PRPF31* |
|  | 23117 | A/C | 82.4/17.6 | 17 | 3 | *PRPF31* |
|  | 23126 | A/C | 84.2/15.8 | 19 | 3 | *PRPF31* |
|  | 23320 | T/C | 87.0/13.0 | 23 | 3 | *PRPF31* |
|  | 23321 | T/G | 83.3/16.7 | 18 | 3 | *PRPF31* |
|  |  |  |  |  |  |  |
| Illumina GA at 10% threshold | 9141 | G/A | 85.5/14.5 | 4558 | 661 | *TFPT* |
|  |  |  |  |  |  |  |
