# Supplementary material for: Ultra High Throughput Sequencing in Human DNA Variation Detection: A Comparative Study on the NDUFA3-PRPF31 Region
Source: PLoS One. 2010 Sep 29;5(9):e13071. doi: 10.1371/journal.pone.0013071 (PMC2947511; doi:10.1371/journal.pone.0013071)
Supplement: Table S3 — SNPs detected after mapping of UHTS untrimmed reads. Black: SNPs detected by using the default threshold for heterozygozity (20%). Red: SNPs detected with a threshold between 10% and 20%. Blue: SNPs with a borderline limit definition of homo-heterozygosity. Green: variants corresponding to the CAAG insertion. Grey shadow: SNPs located in the 2nd long range PCR fragment, showing allelic imbalance. SNPs identified in the tandem repeats are not reported. (0.22 MB DOC) [file pone.0013071.s005.doc]

|  |  |  |  |  | **ROCHE 454** | | | | **ILLUMINA GA** | | | | **ABI SOLiD** | | | |
| --- | --- | --- | --- | --- | --- | --- | --- | --- | --- | --- | --- | --- | --- | --- | --- | --- |
| **GENE** | **Annotated SNPs** | **Validated**  **by**  **Sanger** | **Ref_seq position** | **Ref. allele** | **Alleles** | **Frequencies** | **Coverage** | **Count of 2^nd^ allele** | **Alleles** | **Frequencies** | **Coverage** | **Count of 2^nd^ allele** | **Alleles** | **Frequencies** | **Coverage** | **Count of 2^nd^ allele** |
| *NDUFA3* | rs254260 |  | 450 | T | G | 99.9 | 1897 |  | G | 100.0 | 1632 |  | G | 99.7 | 12814 |  |
| *NDUFA3* | rs254259 |  | 646 | T | T/C | 61.7/38.1 | 575 | 219 | T/C | 62.6/37.4 | 1919 | 718 | T/C | 50.8/49.1 | 8365 | 4110 |
| *NDUFA4* | rs2118214* | yes | 2286 | G |  |  |  |  | G/T | 71.4/28.5 | 2977 | 847 | G/T | 89.2/10.6 | 5450 | 575 |
| *NDUFA3* |  |  | 2476 | G | G/A | 59.4/40.4 | 636 | 257 | G/A | 56.4/43.4 | 3415 | 1483 | G/A | 56.9/43.0 | 21062 | 9066 |
| *NDUFA3* | rs254257 |  | 3268 | A | A/G | 58.0/41.6 | 562 | 234 | A/G | 65.7/34.3 | 2643 | 906 | A/G | 55.1/44.8 | 22847 | 10240 |
| *NDUFA3* |  |  | 3389 | C | C/T | 61.2/38.8 | 619 | 240 | C/T | 61.4/38.5 | 1494 | 575 | T/C | 52.6/47.3 | 20754 | 9813 |
| *NDUFA3* | rs45595133 |  | 3754 | T | G/T | 61.1/38.9 | 18 | 7 | T/G | 52.6/47.2 | 2243 | 1059 | T/G | 70.7/29.0 | 3615 | 1050 |
| *NDUFA3* | rs11878334 |  | 3853 | T | T/A | 63.0/36.2 | 552 | 200 | T/A | 60.5/39.0 | 1650 | 643 | T/A | 60.8/38.9 | 16032 | 6235 |
| *NDUFA3.* (CDS) | rs1061333 |  | 4411 | C | C/T | 52.2/47.8 | 813 | 389 | C/T | 58.9/41.1 | 1207 | 496 | C/T | 81.6/18.3 | 1706 | 312 |
| *TFPT* |  |  | 5224 | G | G/T | 62.5/37.1 | 774 | 287 | G/T | 63.5/36.0 | 1991 | 717 | G/T | 66.5/33.2 | 20108 | 6667 |
| *TFPT* | rs56061812 |  | 5372 | A | A/G | 68.2/31.8 | 676 | 215 | A/G | 64.7/35.3 | 3208 | 1131 | A/G | 52.1/47.9 | 34549 | 16539 |
| *TFPT* | rs2118213 |  | 7341 | G | T | 100.0 | 30 |  | T | 99.6 | 5319 |  | T | 99.3 | 6294 |  |
| *TFPT* | rs12609379 | yes | 7661 | G | *A/G* | *80.8/18.8* | *240* | *45* | *A/G* | *81.4/18.4* | *7484* | *1378* | *A/G* | *86.9/13.0* | *9359* | *1221* |
| *TFPT* | rs254269 |  | 8128 | G | A | 99.1 | 323 |  | A | 99.3 | 6154 |  | A | 99.1 | 10488 |  |
| *TFPT* | rs60371156* | yes | 8337 | T |  |  |  |  | T/A | 87.2/12.5 | 4049 | 505 |  |  |  |  |
| *TFPT* |  | yes | 8564 | C | G/C | 70.1/29.9 | 616 | 184 | *G/C* | *81.2/18.6* | *14914* | *2778* | G/C | 76.7/23.2 | 9674 | 2245 |
| *TFPT* | rs57911619 | yes | 9081 | C | G/C | 60.9/39.1 | 192 | 75 | G/C | 77.7/22.1 | 5641 | 1248 | *G/C* | *82.8/16.9* | *3373* | *571* |
| *PRPF31* | rs4806711 | yes | 13432 | A | G/A | 57.3/42.7 | 503 | 215 | G/A | 70.8/29.1 | 4507 | 1313 | G/A | 70.0/30.0 | 12653 | 3790 |
| *PRPF31* | rs35705606 | yes | 13761 | A | A/G | 74.3/25.7 | 113 | 29 | A/G | 84.4/15.5 | 4462 | 691 | A/G | 85.1/14.9 | 11367 | 1691 |
| *PRPF31* | rs2668836 | yes | 14098 | A | A/C | 69.4/30.6 | 468 | 143 | A/C | 80.8/18.9 | 5588 | 1056 | A/C | 75.7/24.1 | 9431 | 2273 |
| *PRPF31* | rs254277 | yes | 18785 | G | A | 99.6 | 231 |  | A | 98.4 | 1480 |  | A | 96.3 | 3577 |  |
| *PRPF31* |  | yes | 19678 | T | T/G | 59.0/40.9 | 1116 | 456 | T/G | 60.1/39.5 | 2119 | 837 | T/G | 61.8/38.0 | 12695 | 4820 |
| *PRPF31* | rs59977379 | yes | 19705 | A | A/G | 50.0/50.0 | 1051 | 525 | A/G | 58.9/41.1 | 2245 | 922 | G/A | 50.4/49.6 | 14174 | 7025 |
| *PRPF31* |  | yes | 20006 | C | C/T | 54.3/45.7 | 894 | 409 | C/T | 50.5/49.5 | 1846 | 913 | C/T | 50.3/49.7 | 9943 | 4938 |
| *PRPF31* | rs56220912 | yes | 20296 | C | C/G | 57.0/42.9 | 994 | 426 | G/C | 52.4/47.3 | 1066 | 504 | G/C | 69.6/30.3 | 4504 | 1363 |
| *PRPF31* | rs254275 | yes | 20527 | C | G | 99.5 | 409 |  | G | 100.0 | 1510 |  | G | 99.9 | 16010 |  |
| *PRPF31* | rs2303557 | yes | 21338 | T | C | 100.0 | 879 |  | C | 100.0 | 665 |  | C | 99.9 | 3080 |  |
| *PRPF31.* (CDS) | rs1058572 | yes | 21405 | G | G/A | 58.6/41.3 | 1139 | 470 | G/A | 58.7/41.3 | 993 | 410 | A/G | 55.5/44.4 | 8681 | 3858 |
| *PRPF31* | rs56234781 | yes | 21703 | G | A/G | 53.1/46.8 | 1186 | 555 | G/A | 51.0/48.8 | 1435 | 700 | G/A | 63.6/36.4 | 5138 | 1870 |
| *PRPF31* | rs33976447 | yes | 23127 |  | C/A | 86.2/13.8 | 65 | 9 | C/A | 52.7/47.1 | 831 | 391 | C | 99.3 | 539 |  |
| *PRPF31* |  | yes | 23130 |  | G/A | 87.5/12.5 | 295 | 37 | G/A | 69.0/30.9 | 758 | 234 | G | 99.8 | 578 |  |
| *PRPF31* | rs254274 | yes | 23181 | G | A | 99.3 | 584 |  | A | 99.5 | 1315 |  | A | 99.0 | 6044 |  |
| *PRPF31* | rs254273 | yes | 23788 | A | G | 99.8 | 1235 |  | G | 99.6 | 1499 |  | G | 99.9 | 7961 |  |
| *PRPF31* | rs254272 | yes | 23938 | T | T/C | 53.3/46.7 | 1399 | 653 | T/C | 60.8/39.1 | 2092 | 819 | T/C | 64.2/34.2 | 16475 | 5631 |
| *PRPF31* | rs10424816 | yes | 24449 | C | C/A | 58.0/41.9 | 776 | 325 | C/A | 50.4/49.5 | 1847 | 914 | A/C | 59.7/40.1 | 9779 | 3925 |
| *PRPF31* |  | yes | 24876 | T | T/G | 60.1/39.6 | 675 | 267 | T/G | 57.5/42.3 | 2132 | 901 | G/T | 76.0/23.4 | 9364 | 2187 |
| *PRPF31* | rs254271 | yes | 24998 | G | G/C | 57.7/42.3 | 943 | 399 | G/C | 53.2/46.7 | 1582 | 739 | G/C | 65.5/34.2 | 12638 | 4324 |
| *PRPF31* | rs10853869 | yes | 25338 | G | G/A | 56.1/43.8 | 1229 | 538 | A/G | 50.2/49.6 | 3089 | 1533 | G/A | 55.4/44.4 | 20382 | 9048 |
| *PRPF31* |  | yes | 25372 | T | T/C | 83.2/16.5 | 636 | 105 | T/C | 54.7/45.2 | 2239 | 1013 | T/C | 60.2/39.7 | 15970 | 6348 |
| *PRPF31* | rs171703 | yes | 25619 | T | C | 100.0 | 542 |  | C | 100.0 | 604 |  | C | 98.4 | 6125 |  |
| *PRPF31* | rs34990810 | yes | 25871 | C | T/C | 58.5/41.5 | 388 | 161 | T/C | 54.4/45.5 | 794 | 361 | T/C | 70.2/29.7 | 6605 | 1964 |
| *PRPF31* | rs10417221* | yes | 26121 | T |  |  |  |  | C | 98.7 | 226 |  | C | 97.8 | 1963 |  |
| *PRPF31* | rs2668840 | yes | 26152 | A | G | 100.0 | 115 |  | G | 99.6 | 810 |  | G | 99.9 | 12033 |  |
| *PRPF31* | rs667324 | yes | 26177 | G | A | 98.6 | 143 |  | A | 99.6 | 764 |  | A | 99.7 | 10816 |  |
| *PRPF31* | rs2556367 | yes | 26332 | G | G/A | 52.7/47.3 | 245 | 116 | A/G | 51.7/48.3 | 1711 | 826 | G/A | 52.0/48.0 | 10107 | 4847 |
| *PRPF31* | rs2576453 | yes | 26494 | G | A | 100.0 | 295 |  | A | 99.6 | 726 |  | A | 99.5 | 19730 |  |
| *PRPF31* | rs608608 | yes | 26593 | C | G | 99.7 | 298 |  | G | 99.6 | 687 |  | G | 99.6 | 14407 |  |
| *PRPF31* | rs655240 | yes | 26664 | T | *C/T* | *79.7/20.3* | *138* | *28* | C | 99.6 | 978 |  | C | 99.8 | 11411 |  |
|  | rs12150988 |  | 29568 | C | G/C | 53.3/46.7 | 666 | 311 | C/G | 50.6/49.3 | 1901 | 938 | C/G | 54.4/45.4 | 15665 | 7107 |
|  | rs2668838 |  | 30444 | C | T/C | 71.6/28.4 | 95 | 27 | T/C | 51.7/48.1 | 1438 | 692 | C/T | 52.7/47.3 | 38101 | 18015 |
|  |  |  | 30583 | A | A/G | 65.1/34.9 | 350 | 122 | A/G | 61.8/38.0 | 2567 | 976 | A/G | 61.8/38.1 | 32605 | 12422 |
|  | rs254248 |  | 30795 | A | A/G | 70.4/29.6 | 500 | 148 | A/G | 62.6/37.4 | 1769 | 661 | A/G | 54.5/45.4 | 20267 | 9200 |
|  | rs4806715 |  | 30871 | A | C/A | 58.0/41.3 | 460 | 190 | A/C | 62.2/37.7 | 2552 | 961 | A/C | 59.7/40.1 | 34714 | 13926 |
|  | rs2668837 |  | 30965 | C | T/C | 64.1/35.8 | 632 | 226 | T/C | 54.8/45.2 | 2192 | 990 | T/C | 52.0/48.0 | 22426 | 10757 |

*, within homopolymeric sequence.
