# Supplementary material for: Ultra High Throughput Sequencing in Human DNA Variation Detection: A Comparative Study on the NDUFA3-PRPF31 Region
Source: PLoS One. 2010 Sep 29;5(9):e13071. doi: 10.1371/journal.pone.0013071 (PMC2947511; doi:10.1371/journal.pone.0013071)
Supplement: Table S4 — Deletions detected with Roche 454 (false positives), using trimmed reads. The deletion at position 30,672 was also found using the other 2 platforms, likely being the only real small deletion. (0.16 MB DOC) [file pone.0013071.s006.doc]

| **Reference position** | **Reference**  **allele** | **Allele variations** | **Frequencies** | **Coverage** | **Count of 2^nd^ allele** | **Overlapping gene** |
| --- | --- | --- | --- | --- | --- | --- |
| 728 | G | G/- | 64.4/35.6 | 407 | 145 | *NDUFA3, CDS* |
| 775 | C | C/- | 73.5/26.5 | 211 | 56 | *NDUFA3* |
| 782 | C | C/- | 65.1/34.9 | 195 | 68 | *NDUFA3* |
| 1001 | A | A/- | 56.1/37.8 | 180 | 68 | *NDUFA3* |
| 1211 | G | G/- | 51.4/47.5 | 183 | 87 | *NDUFA3* |
| 1217 | G | G/- | 76.4/23.6 | 165 | 39 | *NDUFA3* |
| 1238 | T | - | 42.0 | 143 |  | *NDUFA3* |
| 1238 | TT | -- | 39.2 | 143 |  | *NDUFA3* |
| 1383 | T | T/- | 71.4/28.6 | 84 | 24 | *NDUFA3* |
| 1390 | T | T/- | 78.8/21.2 | 52 | 11 | *NDUFA3* |
| 1550 | T | T/- | 74.3/24.9 | 237 | 59 | *NDUFA3* |
| 2278 | T | -/T | 51.6/43.2 | 190 | 82 | *NDUFA3* |
| 2295 | T | T/- | 76.8/20.6 | 155 | 32 | *NDUFA3* |
| 3679 | C | C/- | 63.6/35.6 | 225 | 80 | *NDUFA3* |
| 3742 | T | T/- | 79.2/20.0 | 125 | 25 | *NDUFA3* |
| 3748 | T | -/T | 48.1/39.4 | 104 | 41 | *NDUFA3* |
| 3892 | T | T/- | 67.8/29.9 | 515 | 154 | *NDUFA3* |
| 4965 | T | -/T | 56.3/38.1 | 446 | 170 | *TFPT* |
| 5763 | G | G/- | 75.0/24.6 | 741 | 182 | *TFPT, CDS* |
| 6354 | C | C/- | 69.9/30.1 | 501 | 151 | *TFPT* |
| 6364 | T | T/- | 61.6/38.0 | 484 | 184 | *TFPT* |
| 6610 | T | -/T | 48.0/47.5 | 1222 | 580 | *TFPT* |
| 7261 | AA | -- | 37.5 | 32 |  | *TFPT* |
| 7261 | A | -/A | 32.4/23.5 | 34 | 8 | *TFPT* |
| 7272 | A | - | 84.4 | 32 |  | *TFPT* |
| 7541 | C | C/- | 76.2/22.9 | 227 | 52 | *TFPT* |
| 7933 | G | G/- | 78.3/21.4 | 429 | 92 | *TFPT* |
| 8326 | A | A/- | 40.0/36.0 | 50 | 18 | *TFPT* |
| 8326 | AA | AA/-- | 31.9/21.3 | 47 | 10 | *TFPT* |
| 8352 | C | C/- | 75.3/24.7 | 85 | 21 | *TFPT* |
| 9442 | A | A/- | 75.3/24.7 | 81 | 20 | *TFPT* |
| 9603 | A | -/A | 44.3/39.2 | 79 | 31 | *TFPT* |
| 9736 | A | -/A | 59.7/37.3 | 67 | 25 | *TFPT* |
| 9877 | A | A/- | 34.6/23.1 | 26 | 6 | *TFPT* |
| 9991 | G | G/- | 79.2/20.8 | 274 | 57 | *TFPT* |
| 10220 | G | G/- | 79.3/20.7 | 188 | 39 | *TFPT* |
| 10561 | A | A/- | 64.7/34.0 | 153 | 52 | *TFPT* |
| 10893 | A | A/- | 71.7/27.4 | 329 | 90 | *TFPT* |
| 11192 | C | C/- | 69.9/29.8 | 302 | 90 | *TFPT* |
| 11233 | A | -/A | 42.2/33.2 | 211 | 70 | *TFPT* |
| 11480 | T | T/- | 59.9/36.3 | 284 | 103 | *TFPT* |
| 12006 | G | G/- | 74.5/25.0 | 200 | 50 | *TFPT* |
| 12702 | CTC | CTC/--- | 75.0/24.7 | 288 | 71 | *TFPT* |
| 13063 | C | C/- | 74.2/25.4 | 524 | 133 | *TFPT* |
| 13513 | T | T/- | 51.9/46.2 | 210 | 97 | *PRPF31* |
| 13519 | T | -/T | 62.0/34.0 | 200 | 68 | *PRPF31* |
| 14563 | A | A/- | 60.2/38.3 | 415 | 159 | *PRPF31* |
| 14716 | A | A/- | 48.9/39.4 | 94 | 37 | *PRPF31* |
| 14735 | A | A/- | 62.8/35.1 | 94 | 33 | *PRPF31* |
| 14858 | G | G/- | 68.8/30.5 | 311 | 95 | *PRPF31* |
| 15305 | AAA | AAA/--- | 23.8/23.8 | 42 | 10 | *PRPF31* |
| 15474 | A | A/- | 77.4/22.0 | 164 | 36 | *PRPF31* |
| 16131 | G | G/- | 77.8/21.9 | 465 | 102 | *PRPF31* |
| 16370 | T | -/T | 37.1/24.3 | 70 | 17 | *PRPF31* |
| 16370 | TT | --/TT | 23.2/20.3 | 69 | 14 | *PRPF31* |
| 16599 | T | T/- | 54.3/41.0 | 427 | 175 | *PRPF31* |
| 17120 | TTT | TTT/--- | 40.0/24.0 | 25 | 6 | *PRPF31* |
| 17289 | T | T/- | 71.8/25.1 | 529 | 133 | *PRPF31* |
| 18396 | T | -/T | 53.2/21.9 | 393 | 86 | *PRPF31* |
| 18767 | A | -/A | 46.2/43.6 | 117 | 51 | *PRPF31* |
| 18925 | AAA | AAA/--- | 21.7/20.8 | 106 | 22 | *PRPF31* |
| 19573 | G | G/- | 70.6/28.9 | 948 | 274 | *PRPF31* |
| 21356 | C | C/- | 74.4/25.5 | 1036 | 264 | *PRPF31* |
| 22089 | C | C/- | 74.7/25.1 | 427 | 107 | *PRPF31* |
| 22302 | C | -/C | 55.7/43.0 | 619 | 266 | *PRPF31* |
| 22309 | C | C/- | 75.5/23.8 | 608 | 145 | *PRPF31* |
| 22961 | A | A/- | 73.2/25.2 | 523 | 132 | *PRPF31* |
| 23294 | C | C/- | 63.1/36.9 | 179 | 66 | *PRPF31* |
| 23314 | T | -/T | 50.0/40.9 | 176 | 72 | *PRPF31* |
| 23763 | G | G/- | 62.9/34.9 | 1135 | 396 | *PRPF31* |
| 23818 | G | G/- | 78.8/20.9 | 1313 | 275 | *PRPF31* |
| 23861 | C | C/- | 66.5/33.1 | 1109 | 367 | *PRPF31* |
| 24122 | C | C/- | 73.7/25.7 | 992 | 255 | *PRPF31* |
| 24741 | A | -/A | 57.1/40.1 | 352 | 141 | *PRPF31* |
| 24747 | A | -/A | 56.3/42.6 | 350 | 149 | *PRPF31* |
| 25184 | G | G/- | 78.6/21.3 | 1277 | 272 | *PRPF31* |
| 25474 | G | G/- | 70.5/29.4 | 797 | 234 | *PRPF31* |
| 25548 | A | -/A | 69.1/30.4 | 573 | 174 | *PRPF31* |
| 25613 | G | G/- | 58.5/40.5 | 1204 | 488 | *PRPF31* |
| 25671 | C | C/- | 63.8/35.9 | 1224 | 439 | *PRPF31* |
| 26120 | CT | -- | 23.7 | 38 |  | *PRPF31* |
| 26121 | T | - | 60.5 | 38 |  | *PRPF31* |
| 26406 | C | C/- | 75.9/23.8 | 294 | 70 | *PRPF31* |
| 26848 | G | G/- | 70.5/29.2 | 359 | 105 | *PRPF31* |
| 28297 | AAA | AAA/--- | 21.1/21.1 | 90 | 19 | *PRPF31* |
| 28297 | AAAA | ---- | 23.5 | 85 |  | *PRPF31* |
| 29326 | T | T/- | 76.2/22.0 | 395 | 87 | *PRPF31, CDS* |
| 30359 | T | T/- | 46.2/20.9 | 91 | 19 |  |
| 30672 | C | -/C | 57.3/42.3 | 494 | 209 |  |
